# Development and evaluation of LLM-based suicide intervention chatbot

**DOI:** 10.3389/fpsyt.2025.1634714

**Published:** 2025-08-05

**Authors:** Xueting Cui, Yun Gu, Hui Fang, Tingshao Zhu

**Affiliations:** ^1^ State Key Laboratory of Cognitive Science and Mental Health, Institute of Psychology, Chinese Academy of Sciences (CAS), Beijing, China; ^2^ Department of Psychology, University of the Chinese Academy of Sciences, Beijing, China; ^3^ The Fifth People’s Hospital of Nanning, Nanning, China

**Keywords:** suicidal ideation, large language model, chatbot, self-help psychological crisis intervention, suicide prevention and intervention

## Abstract

**Introduction:**

Suicide accounts for over 720,000 deaths globally each year, and many more individuals experiencing suicidal ideation; thus, implementing large-scale, effective suicide intervention is vital for reducing suicidal behaviors. Traditional suicide intervention methods are hampered by shortages of qualified practitioners, variability in clinical competence, and high service costs. This study leverages Large Language Models (LLMs) to develop an effective suicide intervention chatbot, which provides early, large-scale, rapid self-help interventions.

**Methods:**

First, according to existing psychological crisis intervention methods, we fine-tuned ChatGPT-4 via prompt engineering to develop a chatbot that promptly responds to the needs of individuals experiencing suicidal ideation. Then, we implemented a self-help web-based dialogue platform powered by this chatbot and conducted the evaluations of its usability and intervention efficacy.

**Results:**

We found that the self-help suicide intervention chatbot achieved high effectiveness and quality in terms of user interface operability, interaction experience, emotional support, intervention efficacy, safety and privacy, and overall satisfaction.

**Discussion:**

These findings demonstrate that the suicide intervention chatbot can provide effective emotional support and therapeutic intervention to a large cohort experiencing suicidal ideation.

## 1 Introduction

Suicide is one of the leading causes of unnatural death globally, with over 800,000 deaths by suicide annually and many more individuals attempting suicide or experiencing suicidal ideation (1, 2). Traditional approaches typically rely on professional practitioners for assessment and treatment, yet these practitioners are often in short supply, have varying qualifications, and incur high costs costs (3, 4). Moreover, current suicide intervention therapies depend on individuals with suicidal ideation to initiate help-seeking, yet many individuals with suicidal ideation are reluctant to seek help and have low motivation to engage with support services. Given the vast population at risk, training enough intervention specialists is highly challenging and extremely expensive. Hence, there is an urgent demand for innovative suicide intervention methods. If more effective intervention services can be delivered efficiently to a large population with suicidal ideation, suicide attempts and deaths could decline markedly. Accordingly, this study concentrates on methods to provide effective intervention services to a large-scale population experiencing suicidal ideation.

Many researchers have developed automated systems to support intervention therapies. Among these, Large Language models (LLMs) are promising for boosting the scalability, accessibility, and personalization of medical interventions (5). LLMs are trained on large-scale text corpora and typically have tens of billions of parameters (6). The emergence of models such as OpenAI’s GPT series, Google’s Bard, and Meta’s LLaMA has created unprecedented opportunities for large-scale language generation and analysis (7). They have excelled in psychological assessments, demonstrating the capacity to infer cognitive states from text (8–11).

Beyond language comprehension, LLMs have proven practical in generative language tasks, notably generative chatbots (12, 13). In the context of suicide intervention, generative chatbots can generate human-like responses to offer fresh insights into suicidal ideation and potentially bolster therapy (14). Previous studies found that, compared with traditional methods, chatbot-based mental health support can lower costs, enhance efficiency, and better safeguard patient privacy (15, 16). LLM-based chatbots can provide psychological support, and offer an innovative technical approach in suicide intervention.

Traditional suicide intervention strategies depend on clinicians who have received specialized training to build a trusting therapeutic alliance with clients and to conduct risk assessment and safety planning via face-to-face or synchronous communication. This person-centered approach emphasizes dynamic decision-making and tailored support (17). By contrast, our AI-driven intervention framework employs prompt engineering and safety filters to simulate empathic dialogue and crisis guidance, enabling real-time detection of crisis signals and standardized responses (18). In practice, compared to conventional models, the AI-driven framework can be rapidly updated and deployed without extensive clinician training, enhancing intervention efficiency and broadening accessibility.

Accordingly, this study used LLMs to develop a chatbot that delivers timely support to individuals experiencing suicidal ideation, enabling early, large-scale, rapid self-help suicide intervention. Specifically, in Study 1 we employed prompt engineering to fine-tune LLM into a suicide intervention chatbot; in Study 2 we implemented and evaluated the web-based self-help chatbot.

## 2 Materials and methods

### 2.1 Suicide crisis intervention

The chatbot is based on three-step ACT model (Assessment–Crisis Intervention–Trauma Treatment) (19), which is specifically tailored to address both acute and traumatic crises.

**Figure 1:**
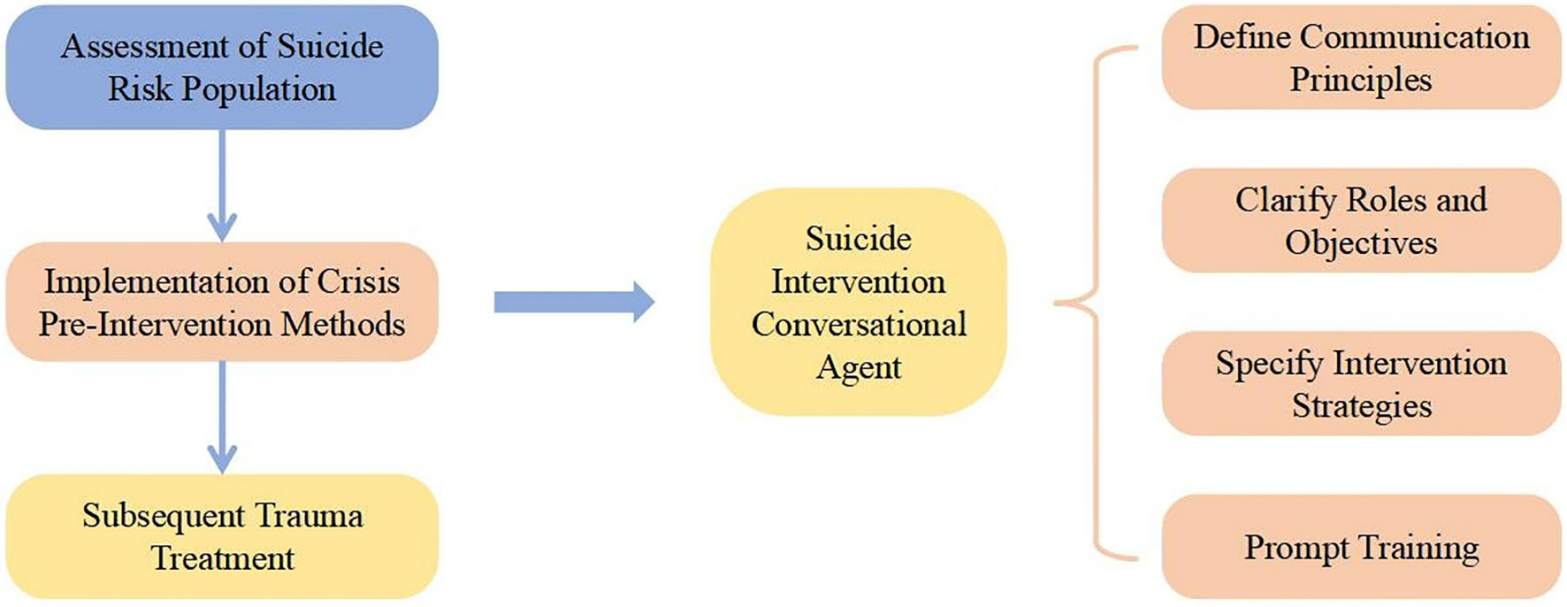
Suicide intervention model and chatbot construction.

Based on previous research, we use a multidimensional suicide risk assessment model to identify high-risk individuals. Then, we provide a self-guided chatbot that delivers targeted therapy as a pre-intervention step. Finally, we offer post-intervention treatment recommendations. The overall intervention procedure is depicted in **Figure 1**.

For individuals at risk of suicide, early intervention depends on effective communication that calms emotions and reduces imminent risks. Accordingly, we trained a LLM to interact with at-risk individuals through a predetermined inquiry process, facilitating targeted suicide intervention.

Currently, LLM is insufficient for targeted tasks as it rarely adapts to specific role. Therefore, a guided training strategy is required so that the model can maintain its designated role and purpose while interacting with individuals at suicide risk. Consequently, we established the framework (shown in **Figure 1**) where the LLM is trained according to specific role requirements and used to conduct suicide crisis intervention following a predetermined process.

### 2.2 Study 1: Developing suicide intervention chatbot

#### 2.2.1 Intervention procedure

**Figure 2:**
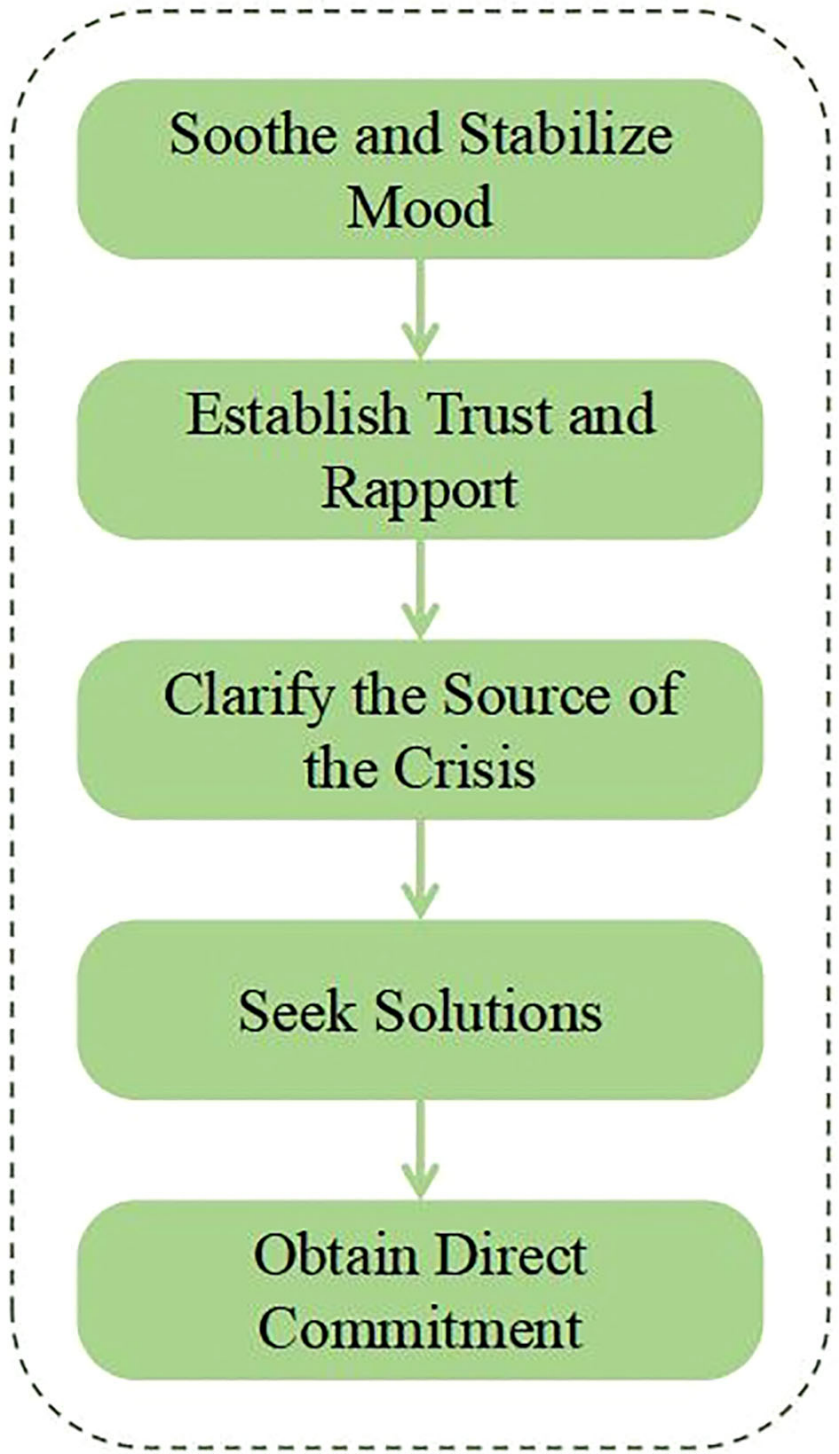
Intervention procedure.

Currently, there is no standardized procedure for crisis intervention. In suicide crises and chat-based approaches, using the traditional six-step crisis intervention method directly is unsuitable (20). Therefore, based on existing psychological crisis intervention manuals, we formulated a general dialogue-based procedure for suicide crisis intervention, as in **Figure 2**.

1. Soothe and Stabilize Mood: Mitigate anxiety through gentle conversation, guided deep breathing, or meditation; ensuring emotional stability, clearer thinking, and reduced impulsive behavior.
2. Establish Trust and Rapport: Clearly articulate a confidentiality commitment (except in urgent safety scenarios), emphasize unconditional acceptance and assure the individual that their thoughts will not be judged.
3. Clarify the Source of the Crisis: Through empathetic inquiry and active listening, thoroughly explore the specific causes and contextual factors that trigger negative emotions or suicidal ideation, and deliver corresponding crisis resolution measures to the large language model.
4. Seek Solutions: Encourage the individual to consider additional problem-solving strategies and collaboratively formulate a practical action plan to effectively manage and stabilize their emotional.
5. Obtain Direct Commitment: Following the development of the coping plan and safety protocols, strive to secure an unequivocal commitment from the individual to adhere to the agreed measures, refrain temporarily from self-harm, and seek assistance when experiencing distress or suicidal ideation.

#### 2.2.2 Prompt training

**Figure 3:**
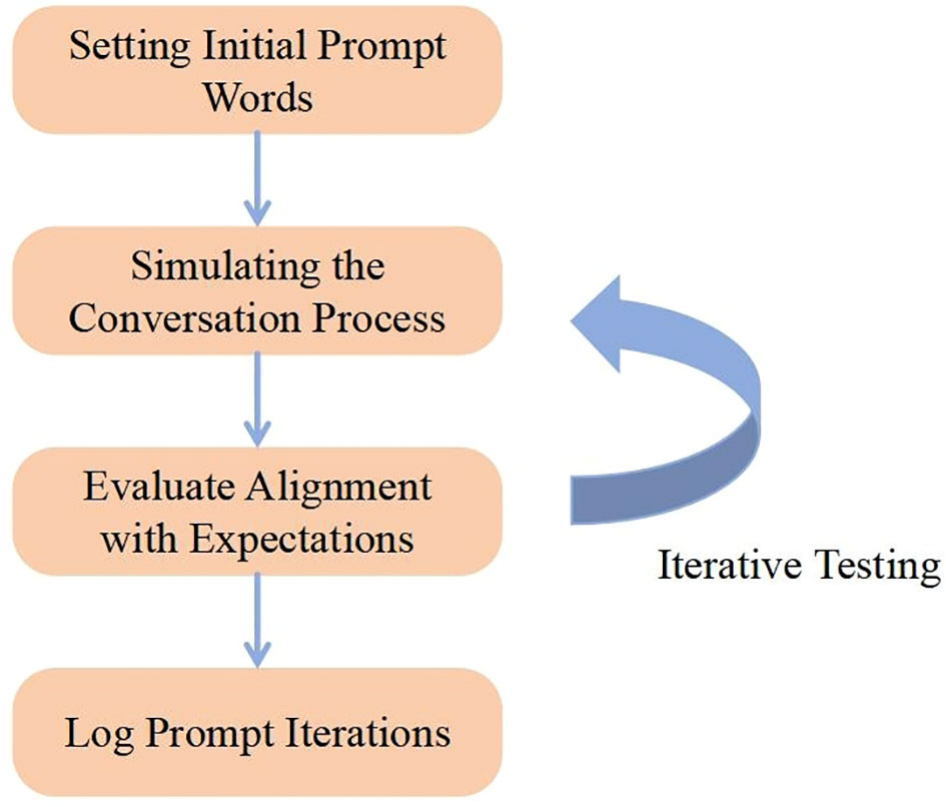
Prompt word training process.

We employ prompt engineering techniques to guide a large language model in performing suicide intervention for at-risk populations, ensuring it follows a designated role, specified objectives, and established principles consistent with the intervention strategy. Initially, we create a set of baseline prompt phrases to ensure the chatbot’s dialogue logic to conform to the pre-established intervention strategy. Next, we iteratively test the model by using the designed prompt phrases and verify whether the outputs meet the expected criteria. Based on the test outcomes, we refine the structure, language, and details of the prompts to optimize the model’s performance, ultimately arriving at appropriate prompt phrases. The overall process is shown in **Figure 3**.

Iterative testing and adjustment are crucial steps in designing the suicide intervention chatbot. This process helps ensure that the content produced by the chatbot affects users in line with our designated intervention strategy, thereby achieving intervention to a certain extent.

We interact with the model using an initial prompt and monitor whether the generated dialogue follows the predefined suicide-intervention procedure. If the output deviates from expectations or contains inappropriate responses, we iteratively refine the prompt’s content, structure, or wording—repeating this cycle until the chatbot consistently follows the specified process.

Each step of the process is as follows:

1. Setting Initial Prompt Words: Using the designed initial prompt, we initiate an opening dialogue with the LLM—for example:

“Right now, I feel like I’m standing on the edge of an abyss. Everything is dark in front of me, and I’m filled with despair. Every morning I wake up asking myself, what’s the point of all this?”

We then observe whether the model’s response conforms to our criteria.

2. Simulating the Conversation Process: By assuming the designated role, gradually reveal the user’s emotional state and needs, guiding the chatbot to identify potential crises and provide real-time intervention and treatment.
3. Iterative Testing: Iterative testing is critical components in the development of a suicide intervention chatbot. This process ensures that the chatbot’s outputs adhere to our designated intervention strategy and effectively influence users. It may necessitate multiple cycles of re-testing with adjusted prompt phrases, each time fine−tuning based on observed responses.
4. Documentation of Iterations: Following each iteration, document which prompt adjustments proved effective and which did not. Such documentation fosters an understanding of prompt designs that yield outputs closely matching expectations and accelerates optimization in future, similar tasks.
5. Integration of User Feedback: We integrate real user feedback into an iterative cycle to identify user needs and concerns, thereby extending the dialogue’s adaptability and scope. Moreover, this feedback confirms whether the prompts are clear and whether the model’s responses address users’ actual needs and emotional experiences.

After multiple rounds of testing and improvement, we developed an optimized prompt sequence that enables the chatbot to assist users effectively by guiding them through the designated suicide-intervention procedure and resolving their concerns. Overall, this feedback-driven improvement process aims to create a suicide-intervention chatbot that respects individual differences, listens nonjudgmentally, shows empathy and equal regard, encourages users, and helps stabilize their emotions.

#### 2.2.3 Parameter settings

While iteratively testing and adjusting the prompt phrases, in order to improve the reliability and appropriateness of the chatbot’s responses, we also need to adjust certain configuration parameters to obtain different prompt outcomes. The parameters are set as shown in **Table 1**, with the following descriptions for each parameter:

1. Temperature: A lower temperature makes the model’s output more predictable, as it picks the most likely words. In contrast, a higher temperature adds variation, leading to more diverse or creative responses. Practically speaking, fact-based Q&A tasks use a low temperature for clear, concise answers, whereas creative tasks like poetry benefit from a higher temperature to generate varied ideas. Since our suicide-intervention chatbot needs to provide stable, empathetic, and structured support, we set a relatively low temperature to reduce unexpected or inappropriate replies.
2. Top−P: Top−P (nucleus) sampling is a probabilistic sampling strategy used in natural language generation. It aims to select coherent and contextually appropriate words while allowing some randomness to encourage creative variation. This method works by choosing from the smallest set of candidate words whose cumulative probability reaches a threshold P (ranging between 0 and 1). For example, with P set to 0.8, the model samples from the most likely words until their combined probability reaches 80%, ignoring less probable options beyond that point. Given the need for precise and targeted dialogue in suicide intervention, setting Top−P at a moderate or slightly lower value helps maintain adequate randomness while ensuring outputs closely align with validated intervention guidelines.
3. Max Length: The max_tokens parameter sets an upper bound on the number of tokens the model generates, preventing overly verbose or off−topic responses. For a suicide intervention chatbot, capping response length ensures messages remain concise enough to sustain user engagement while fulfilling therapeutic objectives.
4. Presence Penalty: The presence penalty is a mechanism to reduce repetition in generated text by lowering the chance of reusing words or phrases that have already appeared. When the presence penalty is set higher, the model is more likely to avoid repeating earlier terms, encouraging more diverse and creative responses. In contrast, a lower or zero presence penalty allows repeated use of the same words, which may result in redundant or less varied output. Because the primary task is to provide reliable, ethically compliant crisis support, it is advisable to maintain a relatively low presence penalty, thereby allowing essential scripted prompts to recur when needed.

**Table 1:** Parameter settings.

Parameter	Value
Temperature	0.5
Top-P	0.8
Max Length	400
Presence Penalty	0.1

### 2.3 Study 2: Implementation and evaluation of the self−help intervention website

#### 2.3.1 Website implementation

After careful evaluation of safety, domain fit, and multilingual support, we selected GPT-4 as the core engine for our suicide-intervention chatbot. GPT-4’s industry-leading safety mechanisms—including rigorous harmful-content filtering and response review—help reduce misleading or triggering statements, making its recommendations more reliable in high-risk scenarios (21).

Next, research shows that GPT-4 produces notably more empathetic responses on emotional-support tasks than earlier models (22, 23). Moreover, GPT-4’s large context window of up to 128K tokens and its 99.9% API uptime support consistent, multi-turn intervention dialogues (21, 24).

Accordingly, we call the GPT-4 API on a Python-based backend for data processing and build the frontend with Gradio—using global variables and the State feature to track sessions and history, storing dialogue logs in a hash map, and deploying via the Gradio server for web access.

**Figure 4:**
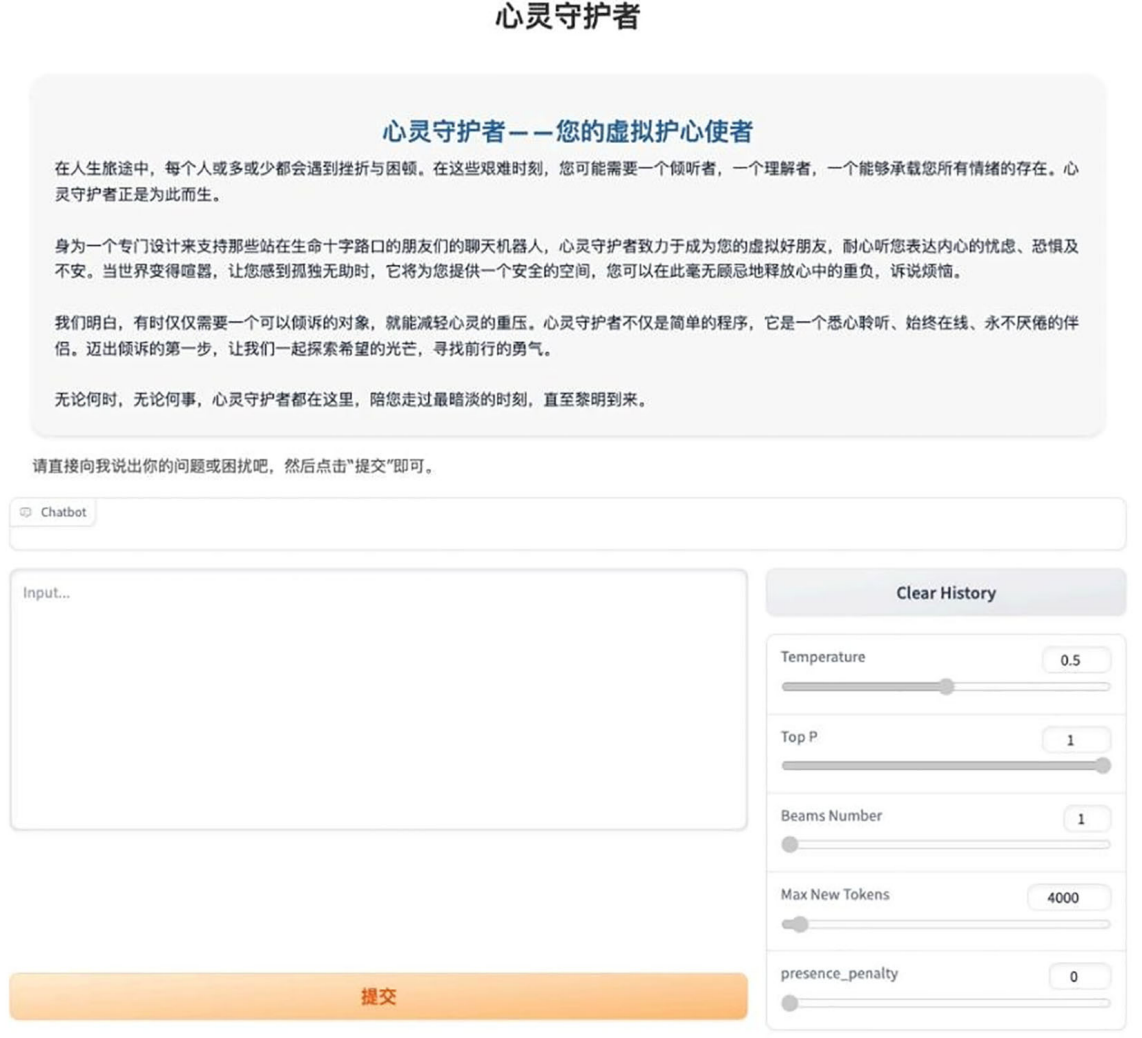
“Mind guardian” chatbot interface.

**Figure 4** illustrates its front−end interface, and we named it “Mind Guardian” to humanize the AI and reduce psychological distance.

In our system design, participant privacy and anonymity are fully maintained. All session data are end-to-end encrypted, and key identifiers (e.g., IP address, device IDs) are obfuscated using random encoding. The backend stores only anonymized aggregate statistics for analysis, ensuring that no personally identifiable raw data are retained. User interaction runs entirely in anonymous mode—no registration or real-identity is required—and the interface reminds participants at startup and during the conversation to avoid sharing names, contact details, or other sensitive information. Finally, an input-validation check prevents users from entering nonessential fields.

#### 2.3.2 Questionnaire survey

To evaluate its performance, we first conducted an expert assessment. Twenty psychology professionals participated in a complete communicative therapy session with “Mind Guardian”. The procedure had three phases:

1. Dialogue: Experts logged into the chat platform, reviewed the study’s tasks and objectives, and engaged with “Mind Guardian” using guided prompts. The session began with reference prompts, during which participants gradually revealed the user’s emotional state and needs, guiding the chatbot to detect potential crises and assess its response capabilities.
2. Evaluation: Predefined evaluation criteria were applied to each chatbot response. Experts rated the chatbot on its accuracy in recognizing emotions and suicide risk, as well as the timeliness and relevance of its interventions.
3. Questionnaire Survey: Following the interactive session, participants completed an eight-statement, six-dimension questionnaire adapted from established mental health assessment tools (shown in **Table 2**). The survey captured overall chatbot performance, recorded any technical issues, and solicited suggestions for improvement.

**Table 2:** Questionnaire design.

Dimension	Number	Items
User Interface and Operability	1	I consider “Mind Guardian” easy to operate, with a user−friendly interface.
Interaction Experience	2	My dialogue with “Mind Guardian” was natural and smooth.
	3	I believe “Mind Guardian” accurately understood my expressions.
Emotional Support	4	I could feel “Mind Guardian”‘s emotional support (e.g., empathy).
	5	After chatting with “Mind Guardian,” I could calm my emotions.
Intervention Effectiveness	6	I believe the suggestions provided by “Mind Guardian” were helpful to me.
Safety and Privacy	7	I felt that my privacy and data security were adequately safeguarded when interacting with “Mind Guardian.”
Overall Satisfaction	8	Overall, I am highly satisfied with the performance of “Mind Guardian.”

## 3 Results

Detailed results of the self-help suicide intervention chatbot’s performance across six dimensions are summarized in **Table 3**. Overall, expert evaluations demonstrated high effectiveness and quality, particularly in user interface and operability, which received the top scores despite minor discrepancies among raters. Interaction experience also garnered elevated ratings, with a low standard deviation indicating consistent perceptions of engagement quality. Moreover, the emotional support dimension scored near 6 on a 7 - point scale, suggesting strong affective support capabilities. While intervention effect evaluations were generally positive, the larger variance points to differences in experts’ judgments, likely influenced by individual assessment criteria.

**Table 3:** Survey statistical results.

Dimension	Number	M	SD	Max/Min
User Interface and Operability	1	6.33	0.82	7/5
Interaction Experience	2	6.13	0.74	7/5
	3	6.27	0.59	7/5
Emotional Support	4	6.00	0.75	7/5
	5	5.93	0.59	7/5
Intervention Effectiveness	6	6.13	0.92	7/5
Safety and Privacy	7	5.93	0.88	7/4
Overall Satisfaction	8	6.00	0.65	7/5

The safety and privacy dimension scored slightly lower and exhibited a relatively high standard deviation, reflecting reasonable concerns raised by at least one expert regarding data security in mental health contexts. Given the high - risk, sensitive nature of suicide intervention populations, such privacy concerns are not only understandable but essential to address. To mitigate these concerns, we will implement multiple privacy−preserving technical measures—most notably, anonymization of user data and secure backend data purging at regular intervals—to prevent unauthorized information leakage. Furthermore, all data handling and privacy safeguards will be transparently communicated to users before any interaction, ensuring informed consent and fostering user trust. Finally, the overall satisfaction score of 6, coupled with a low standard deviation, indicates a favorable consensus regarding the chatbot’s utility and acceptability.

In summary, the self-help suicide intervention chatbot received positive evaluations from psychology professionals, confirming its capacity to deliver emotional support and facilitate intervention efforts for at-risk individuals.

## 4 Discussion

### 4.1 Main findings

In LLM-based suicide prevention research, enhanced suicide risk identification and clinical decision support tools represent the predominant clinical applications, with researchers leveraging LLM to improve suicide risk identification and prediction (14). However, suicide risk detection alone is insufficient; it needs to be integrated with effective, scalable intervention measures (25).

Grounded in suicide crisis intervention methods, our study employs LLM to develop and evaluate a suicide intervention chatbot. We demonstrate its potential as an innovative digital mental health support tool that delivers rapid, large-scale, self-guided interventions, thereby helping to mitigate shortages of qualified practitioners, variability in clinical competence, and high service costs.

The suicide intervention chatbot excelled across multiple dimensions of intervention effectiveness, particularly in user interface operability and accurate comprehension of user expressions, which is consistent with research that emphasizing the importance of these factors in digital mental health interventions (26–28).

Moreover, positive feedback on emotional support and intervention effectiveness suggests that the suicide intervention chatbot can facilitate a clearer understanding of users’ distress through specific, structured interventions and offer more targeted recommendations (29). This aligns with Vlaescu et al. (30), who found that technology-based mental health interventions can help users better understand treatment content and engage in self-management.

These results can be attributed to our comprehensive evaluation framework based on established intervention strategies and rigorous prompt engineering. They represent a significant advancement in AI-driven mental health support tools and demonstrate their potential as safe, effective, large-scale digital interventions.

Research indicates that a significant proportion of individuals who die by suicide have not engaged with formal mental health services, often due to limited access to care and feelings of shame about seeking help (31). Our suicide intervention chatbot demonstrates the feasibility of leveraging artificial intelligence to deliver self-guided mental health interventions. Although it cannot fully replace professional psychotherapists, in settings of resource scarcity or when timely clinician access is unavailable, it serves as an adjunctive tool—especially for individuals uncomfortable with face-to-face therapy or experiencing shame-related barriers—by providing anonymous, on-demand self-help interventions. Indeed, preliminary evidence suggests that LLMs are acceptable in supportive mental health contexts: 78% of individuals reported willingness to use ChatGPT for self-diagnosis or symptom management (32). Moreover, some users indicated a preference for disclosing information to a virtual agent, as it reduces fear and self-presentation concers and facilitates emotional expression (33).

However, research has underscored significant challenges and areas for improvement in AI-driven mental health support tools—especially in safety and privacy. Therefore, developing an ethically robust suicide intervention chatbot is of great importance. Our evaluation of the suicide intervention chatbot indicates that users have significant concerns regarding its safety and privacy. This reflects a central ethical dilemma about balancing potential user safety with individual’s right to privacy in LLM-based suicide prevention research. Kang and Hong (34) removed user registration and personal data collection, so the chatbot depends entirely on the LLM to provide personalized experiences within individual sessions without retaining user-specific information across conversations. While this design enhances data security, it concurrently limits the chatbot’s longitudinal personalization capabilities. Future research could explore advanced techniques—such as federated learning and differential privacy—to enable enhanced personalization without compromising user privacy. Furthermore, it is essential to establish comprehensive privacy policies to safeguard users’ mental health data.

Moreover, given the ethical and safety concerns in high-risk scenarios, we used expert evaluation rather than pre-post testing with real users. Directly testing an unvalidated system on individuals at suicide risk could cause secondary harm; thus, we engaged mental-health professionals in controlled role-play inputs to examine intervention logic and identify potential vulnerabilities, ensuring the model’s responses remain safe and compliant.

This approach aligns with best-practice guidelines for digital mental-health safety assessments (35, 36) and follows the staged validation pathway outlined in the WHO’s digital interventions framework (37), which stipulates that technical functionality and ethical-risk assessments by experts must precede broader real-world user testing.

In the future, under ethics-committee oversight, we will conduct limited-scale pre-post testing with real users using standardized measures of emotional change, thereby providing stronger empirical support for clinical application.

In summary, the suicide intervention chatbot, as a supplementary mental health resource, represents a promising approach in digital mental health interventions. Although the work remains ongoing, its applications and developmental prospects are highly encouraging. However, it must be emphasized that, as a complement to traditional mental health services, the suicide intervention chatbot cannot replace professional mental health treatment, particularly in high-risk cases.

### 4.2 Strengths and limitations

A key strength of this study is the utilization of advanced LLM technology to develop an effective mental health support tool that can facilitate large-scale suicide intervention.

Nevertheless, the study has several notable limitations. Specifically, the efficacy of individual psychological interventions may be affected by user-specific factors such as cognitive styles and personality traits (38). For example, some users may be predisposed to skepticism toward technology or may prefer human interaction. These factors may reduce their acceptance of chatbot-based psychological intervention and, in turn, diminish intervention effectiveness. Future research could investigate more diverse, user-tailored chatbot services to better accommodate individual characteristics.

Moreover, reliance on the GPT API imposes technical limitations on the study (39), as the chatbot’s performance depends on the underlying model, which may carry inherent biases and limitations. In natural language generation, a hallucination occurs when the model outputs content that looks factual but has no real basis in the data (40). To address this, future work could integrate retrieval-augmented generation (RAG), which use external knowledge sources to check facts in real time and thus cut down on hallucinations. This approach would enhance response accuracy and usability while keeping intervention safety intact. Furthermore, dependence on an external API raises concerns about data privacy and long-term system sustainability.

Although GPT-4 performed well overall in our expert evaluation—showing that current prompt designs and model capabilities meet general self-help needs—it still faces challenges with localized psychological expressions. In particular, GPT-4 sometimes struggles to capture deep connotations and cultural contexts. Therefore, future work should optimize Chinese-language prompt design and conduct multi-model comparisons by introducing local LLMs such as Qwen and DeepSeek, aiming for more precise and reliable intervention effectiveness for culturally specific expressions.

These limitations highlight the importance of large-scale, longitudinal studies to comprehensively evaluate the effectiveness and generalizability of LLM-based chatbots.

## 5 Conclusion

In this study, we developed a chatbot for suicidal ideation intervention by crafting tailored prompts and fine-tuning LLM. The results indicate that the suicide intervention chatbot can, to some extent, provide effective emotional support and therapeutic intervention to people experiencing suicidal ideation. Not only do we introduce an effective large-scale self-help psychological crisis intervention approach—with notable advantages in technological initiative, non-contact delivery, cost, and efficiency—but we also highlight the potential of artificial intelligence to support mental health care.

## Data Availability

The original contributions presented in the study are included in the article/Supplementary Material. Further inquiries can be directed to the corresponding authors.
